# Computational analyses decipher the primordial folding coding the 3D structure of the beetle horn

**DOI:** 10.1038/s41598-020-79757-2

**Published:** 2021-01-13

**Authors:** Keisuke Matsuda, Hiroki Gotoh, Haruhiko Adachi, Yasuhiro Inoue, Shigeru Kondo

**Affiliations:** 1 Pattern Formation Laboratory, Graduate School of Frontier Biosciences, Osaka University, Suita, Osaka 565-0871 Japan; 2 Osaka University Hospital, Osaka University, Suita, Osaka 565-0871 Japan; 3 Ecological Genetics Laboratory, Department of Genomics and Evolutionary Biology, National Institute of Genetics, Mishima, Shizuoka 411-8540 Japan; 4 Department of Micro Engineering, Kyoto University, Kyoto, 615-8540 Japan

**Keywords:** Biophysics, Computational biology and bioinformatics, Developmental biology

## Abstract

The beetle horn primordium is a complex and compactly folded epithelial sheet located beneath the larval cuticle. Only by unfolding the primordium can the complete 3D shape of the horn appear, suggesting that the morphology of beetle horns is encoded in the primordial folding pattern. To decipher the folding pattern, we developed a method to manipulate the primordial local folding on a computer and clarified the contribution of the folding of each primordium region to transformation. We found that the three major morphological changes (branching of distal tips, proximodistal elongation, and angular change) were caused by the folding of different regions, and that the folding mechanism also differs according to the region. The computational methods we used are applicable to the morphological study of other exoskeletal animals.

## Introduction

The exoskeleton of arthropods is made of a single sheet of cuticles whose three-dimensional undulations determine body shape. How are the cuticle undulations made? Arthropods grow discontinuously by molting. Because the new cuticle is made directly under the existing one, its shape is similar to that of the previous cuticle. However, it is not entirely the same. This is because the new cuticle has folds before molting, which change their shape as they unfold during molting. For example, Tajiri found that in *Drosophila*, epithelial cells actively crease the cuticle, and that the direction of these creases is responsible for the morphological changes that occur from larvae to pupae^1^. Therefore, elucidating the laws connecting the folding of the cuticle to the unfolded 3D morphology is very important.

The imaginal primordia of holometabolous insects are composed of a single sheet of epithelial cells with dense furrows. For example, the leg discs of *Drosophila* are composed of a single-layered bag with concentric folds. During the pupal stage, they unfold to change the flat shape of the disc into the cylindrical shape of the leg^2,3^. However, examining the relationship between folding patterns and the morphological changes that result from unfolding is not straightforward, because unfolding is not the only factor contributing to transformation. The unfolding progresses slowly in the pupa, during which other cellular phenomena that change the shape of the cell sheet (e.g., cell division, cell rearrangement, and cell transformation) also occur extensively^4–6^. Therefore, elucidating the exact contribution of the folds to 3D morphogenesis remains challenging.

One suitable model system to study the contribution of folding to 3D morphogenesis is the horn of the Japanese rhinoceros beetle, *Trypoxylus dichotomus*. During beetle metamorphosis from larva to pupa, horns of approximately the same size as those of adult beetles emerge. The pupal horn primordium has a very complex folding pattern and is located under the larval head capsule. During pupation, the folded primordium expands like a balloon very quickly due to the pressure of the hemolymph, forming a four-branched horn. In our previous study^7^, we used serial cross-sectional images of the Japanese rhinoceros beetle horn primordia to reconstruct their 3D mesh on a computer. The 3D mesh transformed into a nearly perfect pupal horn shape when pressure was applied from the inside by a computer simulation (Supplementary Figure 1). Thus, we concluded that no factors other than the unfolding of the folds are involved in the transformation from primordium to pupal horn. In other words, the 3D morphology of the horn is encoded in the folding of the primordium.

To understand how the 3D morphology of the horn is developed, it is necessary to decipher the relationship between folding and 3D morphology. However, it is difficult to conduct such an experiment in vivo. To investigate the contribution of the folding to the resulting horn shape, it is necessary to identify which position in the primordium corresponds to a given position on the pupal horn. Although previous studies^8,9^ described the positions in the primordium corresponding to the distal tip of the horn, there are no studies providing an accurate and comprehensive correspondence. Moreover, since there is currently no way to artificially manipulate the folding pattern, it is impossible to prove the contribution of folding to morphological changes through experiments. Unfolding of furrows is a purely physical process, so biological experiments can be substituted for calculations. In our previous study^7^, we partly investigated how the specific pattern of the furrows transform in 3D. However, it is only in each local area, and how the deformation of each part affects the morphology of the entire horn has not been studied. To decipher the meaning of the furrow pattern, the calculation needs to be done in a more systematic way for all areas. In the following sections, we describe the newly developed methods used to decipher the folding pattern and explain the laws whereby the 3D morphology of the horn is embedded in the folding patterns.

## Results

### Methods for analyzing the furrows in horn primordia

In our previous work, we reconstructed a three-dimensional mesh of horn primordia from serial cross-sectional images of actual horn primordia samples acquired by the CoMBI method. When pressure was applied to the 3D mesh from the inside, the folds extended and their shape transformed into the pupal horn shape. Here, we analyzed the relationship between the folding patterns and transformation using the 3D mesh model and computer simulation in the ways described below.

**Figure 1:**
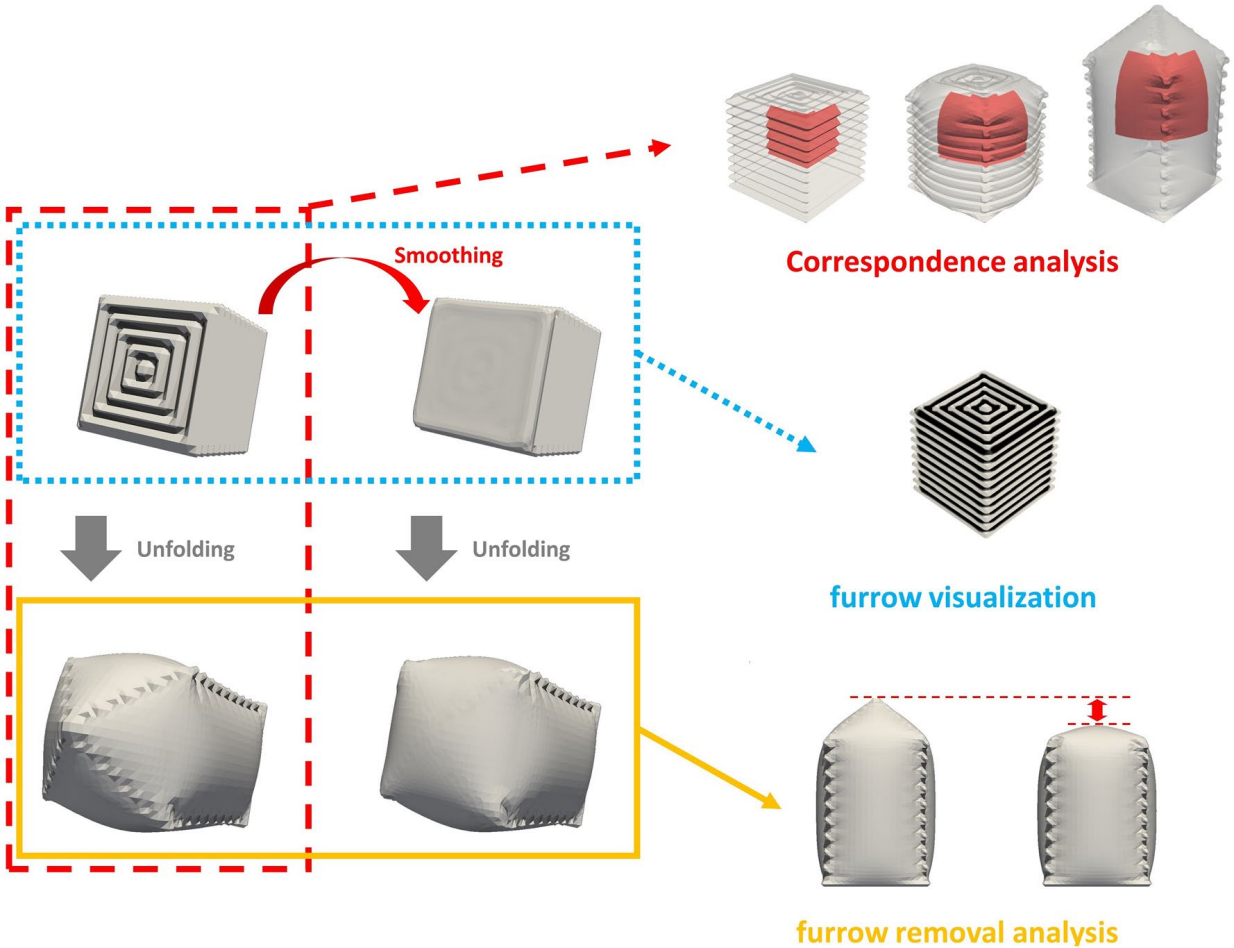
Methods for analyzing furrows and transformations. In Matsuda et al.^7^ reconstructed a three-dimensional mesh of horn primordia that can transform into the pupal horn shape through computer simulation of applying pressure to the 3D mesh from the inside. In this work, we developed three methods to study the relationship between folding patterns and transformation, using the unfolding simulation together with smoothing methods. The first one is the correspondence analysis (red broken lines), in which the position in the folded primordia corresponding to a given position in the unfolded primordia can be easily identified. The second one is furrow visualization (blue dotted lines). The first step of the operation was smoothing the mesh, and then, by comparing the original mesh and the smoothed mesh, the ridges and valleys were colorized. We used the HC-modified Laplacian smoothing algorithm as a smoothing filter. The third method is the furrow removal analysis (yellow solid lines). The first step of the analysis also consisted of smoothing the mesh. Then, the smoothed mesh was expanded in the computer simulation. Because smoothing algorithms can make the furrows at a particular position shallower, the contribution of specific furrows to transformation can be understood by comparing the original mesh with the smoothed mesh after unfolding (red double-headed arrow). The details of each algorithm are provided in the supplementary material.

First, a three-dimensional mesh was used to determine the positional correspondence between the pupal horn and horn primordium. Because the surfaces of biological samples of horn primordia have very complex nested structures with macro folds and micro furrows, it is very difficult to identify the correspondence of a given position in the folded primordium after deployment. However, using the 3D mesh model facilitates this task. After marking some regions of the expanded 3D mesh, the physical simulation was rewound to show exactly which positions in the folded primordium corresponded to those regions. (Fig. 1, red broken lines). This analysis is referred to as “correspondence analysis” in the following sections.

Second, the furrow patterns on the 3D mesh model were visualized. Micro furrows are located on the surface of complex three-dimensional macro shapes. Therefore, it is not possible to observe all furrow patterns even if the light comes from a single direction to create a shadow. It is also necessary to know where and in what direction the furrows run in the structure both before and after deployment. To solve these problems, we used smoothing algorithms, which were originally used to reduce noise from the data and create a smooth mesh surface. By comparing the position of the surface before and after smoothing, we were able to color the ridges and valleys of the furrows (Fig. 1, blue dotted line). In the following sections, we refer to this operation as “furrow visualization.” By using furrow visualization together with the correspondence analysis, it was also possible to colorize the unfolded primordium depending on whether the positions were ridges or valleys when folded.

Third, we used the mesh and applied the smoothing algorithm to investigate how the furrows at each position contribute to transformation. The algorithm can make the furrows at a specific position shallower. Thus, the contribution of specific furrows to transformation can be understood by comparing the original mesh with the operated mesh after unfolding (Fig. 1, yellow solid line). In the following sections, we refer to this operation as “furrow removal analysis.”

The details of each algorithm are provided in the supplementary material.

### Overview of morphological changes

**Figure 2:**
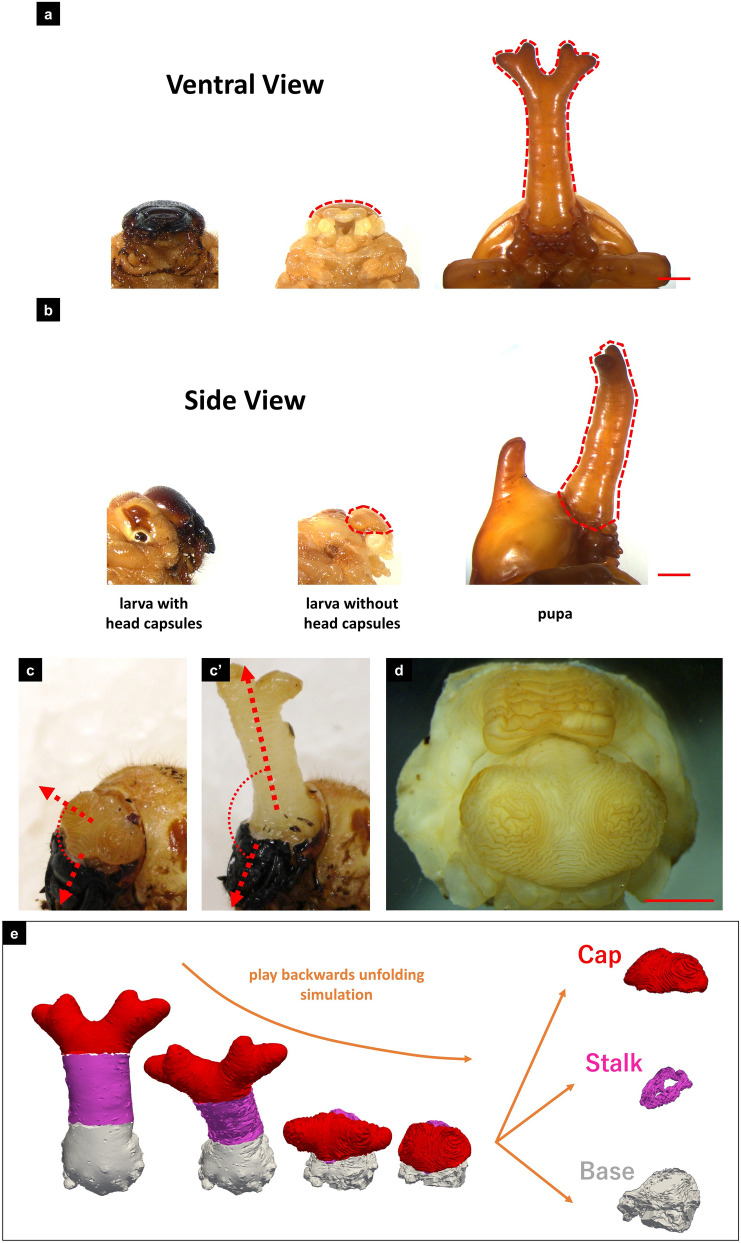
The transformation and division of horn primordia. (**a**,**b**) Comparison of larvae (left), peeled larvae (center), and pupae (right). (**a**) Ventral view. (**b**) Lateral view. Horn primordia look like domes, while pupal horns have a four-branched shape (red broken lines). The proximodistal size of a pupal horn was 6 times as big as that of a horn primordium, while the dorsoventral size changed little (areas surrounded by red broken lines). The scale bars are 5 mm. (**c**,**c′**) Forced expansion treatment^7^. In this treatment, we pushed the larval abdomen to expand horn primordia after removing the larval head capsules to observe only the transformation of horn primordia. The orientation of pupal horns was dorsally declined compared to that of primordia, as the mandibles were used as a standard (details on the definition of the orientation are given in the supplementary material). (**d**) Horn primordia. Complex furrow patterns were seen on the primordia. The scale bar is 3 mm. (**e**) The unfolded horn primordia were divided into 3 regions based on the cylindrical structure of the middle part. We named these parts cap (red), stalk (pink), and base (white), respectively. The correspondence analysis identified the areas in the folded horn primordia corresponding to each region.

First, we compared the external shapes of larvae, peeled larvae (larvae after removal of the outermost exoskeleton), and pupae (Fig. 2a,b). The proximodistal length of the pupal horn was about six times longer than that of a horn primordium, while the lateral width and dorsoventral length were almost unchanged (Fig. 2a,b). In addition to length change, a four-branched structure not present in the primordia appeared in the pupal horn (Fig. 2a). Next, we performed a forced expansion treatment to lessen the effect of transformation in parts other than horn primordia (Fig. 2c,c′). In the treatment, we pushed the larval abdomen to expand horn primordia after removing the larval head capsules^7^. This treatment, in which the mandibles were used as a reference, revealed that the angle of the pupal horn was dorsally declined compared to that of the primordia (details on the definition of the orientation are given in Supplementary Figure 2).

Thus, the transformation includes three types of changes: shape, length, and angle.

### Division of the primordia into three regions

Next, we studied the relationship between the folding of horn primordia (Fig. 2d) and the morphological changes described above. To study this, we divided the horn primordia into three regions likely to contribute to each of the morphological changes (Fig. 2e). The distal region contained four branched tips. This region was named “cap.” The middle region following the cap was cylindrical with a nearly constant radius. This part was named “stalk.” The proximal region was named “base.” This region connected the horn to the body. The top and bottom edges of the cylindrical part were set as the boundaries.

Each corresponding region in the folded primordia was retrospectively identified in the correspondence analysis (Fig. 2e and Supplementary Movie 1), which allowed us to observe how each region had been transformed separately. The cap, which creates a four-branched structure after unfolding, was shaped like a mushroom umbrella with large overhangs on the left and right sides of the primordia (Supplementary Movie 2). The stalk was very flat and complexly folded in the primordia. This indicated that elongation along the long axis mainly depends on this region (Supplementary Movie 3). The folded shape of the base region was rather flat, and its distal boundary ran almost parallel to its proximal boundary. However, after unfolding, the two boundaries became almost perpendicular to each other (Supplementary Movie 4). From this, it can be inferred that the angular change during transformation depends mainly on this region.

In the following sections, we examine in detail how the deployment of the folding contributed to the transformation of each region.

### Structure of the base

**Figure 3:**
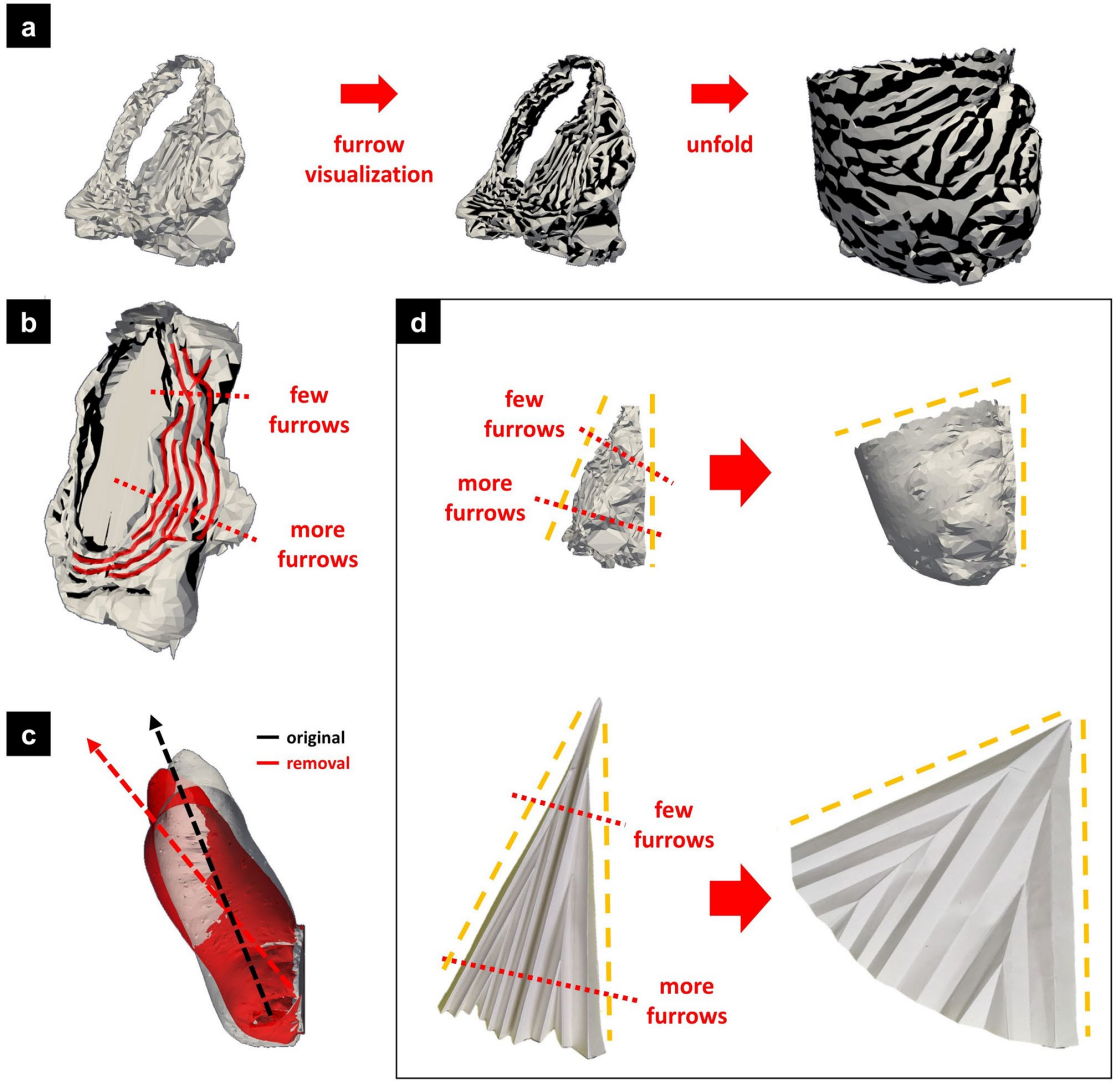
The furrow pattern in the base turns the horn dorsally. (**a**,**b**) Furrow visualization of the base. The unfolding simulation was performed after furrow visualization (**a**). The visualization showed that more furrows were arranged on the ventral side than on the dorsal side. (**b**) Simplest example of the furrow pattern. (**c**) Furrow removal analysis of the base. When the furrows were made shallower, the dorsally turning ratio became smaller (red arrow) than the original (black arrow). (**d**) Contribution of the furrow pattern to transformation. Since the ventral side had more furrows than the dorsal side, the enlargement ratio of the ventral side was much bigger than that of the dorsal side. The difference turned the orientation of the horn dorsally. The upper lane shows the transformation of the base and the lower lane shows the transformation of the origami model.

The base connects the horn to the body. As mentioned above, during the transformation from horn primordium to pupal horn, the overall orientation changes to the dorsal side, to which the unfolding of the furrows in this region should contribute. First, we visualized the furrow pattern in this region using the furrow visualization method (Fig. 3a,b). Apparently, the ventral side had more furrows than the dorsal side (the simplest example is shown in Fig. 3b), which could be the cause of dorsal bending. Second, we performed a furrow removal analysis (Fig. 3c and Supplementary Figure 3a,a′). When all the furrows in the base were made shallower (Supplementary Figure 3a′), the orientation of the unfolded horn changed to the ventral side (Fig. 3c), suggesting the contribution of the furrows in this region to dorsal bending. According to these results, the base has more furrows on the ventral side than the dorsal side, which turns the orientation of the horn dorsally (Fig. 3d).

### Structure of the stalk

**Figure 4:**
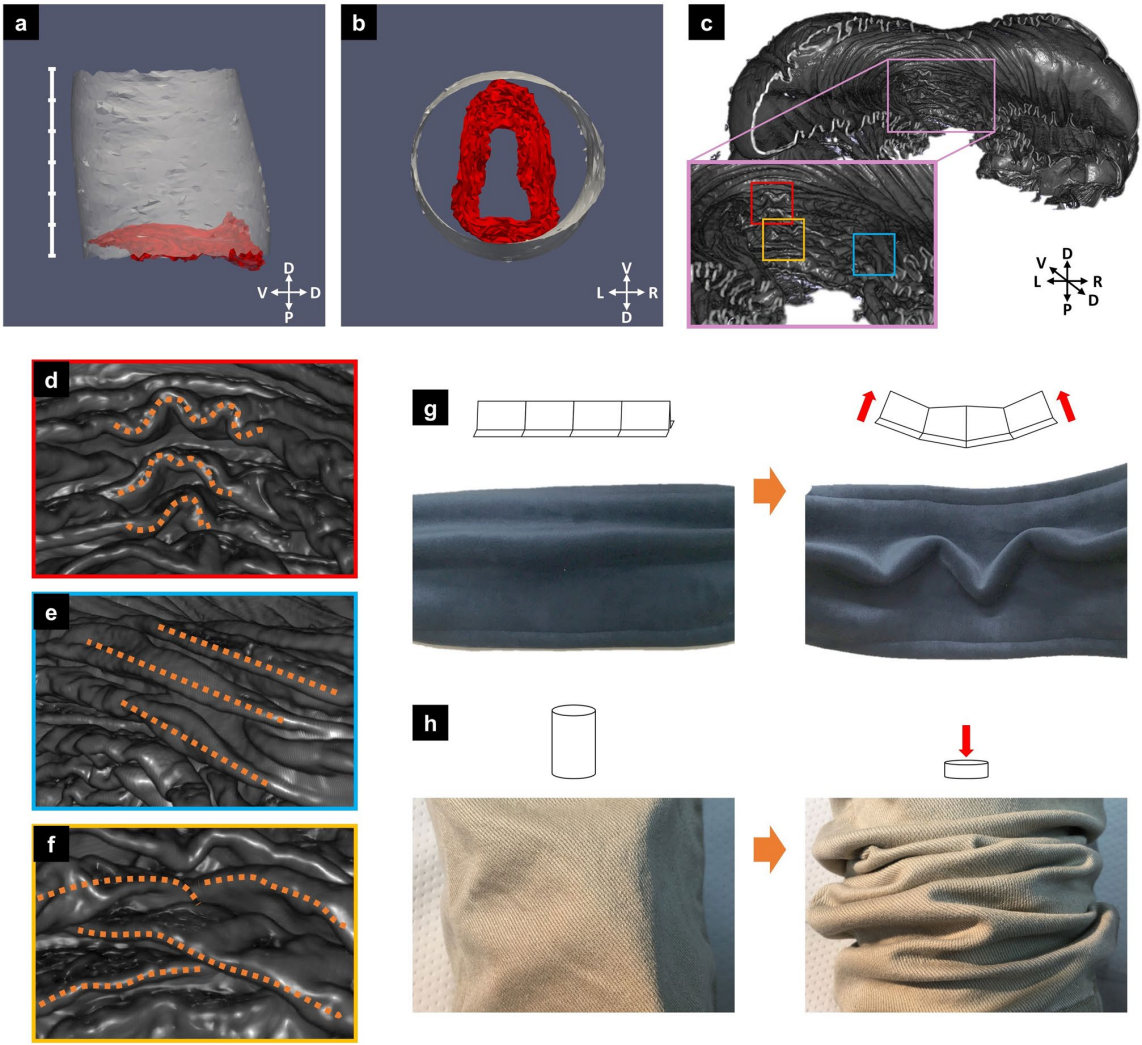
The folding in the stalk has features suggestive of passive formation. (**a**,**b**) Comparison of unfolded and folded stalks after adjusting the orientation. (**a**) The view from the left side revealed that the height of the unfolded stalk (white mesh) was about six times that of the folded stalk (red mesh). (**b**) Viewed from the top side, the folded stalk had an elongated shape that was flattened along the left–right axis. On the other hand, the dorsoventral length of the folded stalk was almost the same size as that of the unfolded stalk. (**c**) Observation of horn primordia from the inside with micro-CT. The pink window is the magnified view of the stalk. (**d**–**f**) Folding patterns seen in the stalk. Each colored window corresponds to the colored window in (**c**). (**d**) Wavy folding. (**e**) Straight parallel folding. (**f**) Zigzag folding. (**g**) The wavy folding (**d**) is formed passively by bending the sheet with folds. (**h**) The zigzag folding (**f**) is formed passively by longitudinal pressure.

As described above, the stalk was the region that mainly contributed to the elongation of the horn. We first compared the stalk region before and after unfolding (Fig. 4a,b; after adjusting the orientation). The proximodistal length of the unfolded stalk was approximately six times longer than that of the folded stalk (Fig. 4a). Seen from the top side, the folded stalk had an elongated shape that was flattened along the left–right axis (Fig. 4b). However, the dorsoventral length of the folded stalk was almost the same as that of the unfolded stalk (Fig. 4b).

Next, we observed the stalk region using a CT scan (Fig. 4c). Because the shape of the unfolded stalk was a cylinder, the furrows in this area did not create a specific three-dimensional shape (because the Gaussian curvature of a cylinder is zero) but contributed to the elongation of the cylinder along the proximodistal axis. Therefore, the direction of folding in most of the stalk region was parallel to the circumference of the cylinder (Fig. 4e). However, if the direction of every fold was parallel to the circumference of the cylinder, like an accordion (Supplementary Figure 4a), the unfolded shape was still wavy (Supplementary Figure 4b). Thus, on the ventral and dorsal sides of the stalk (especially on the ventral side), two more complicated patterns were observed. One consisted of wavy folds (Fig. 4d) and the other consisted of zigzag folds (ridges/valleys alternately coming from the left and right side, Fig. 4f). The zigzag fold, also known as a Yoshimura fold^10^, is a famous folding pattern that inevitably arises when a cylinder-like plane is compressed in the axial direction (Fig. 4h). Because the stalk is surrounded by the cap and base, the growth of a cell sheet in the axial direction is thought to result in relative longitudinal compression, causing the zigzag folds. A wavy fold also appeared autonomously when a sheet with folds was bent (Fig. 4g). Since the lateral tips of the cap occupy both the left and right spaces beside the stalk, the growing cap is thought to exert bending forces on the ventral side of the stalk.

### Structure of the cap

**Figure 5:**
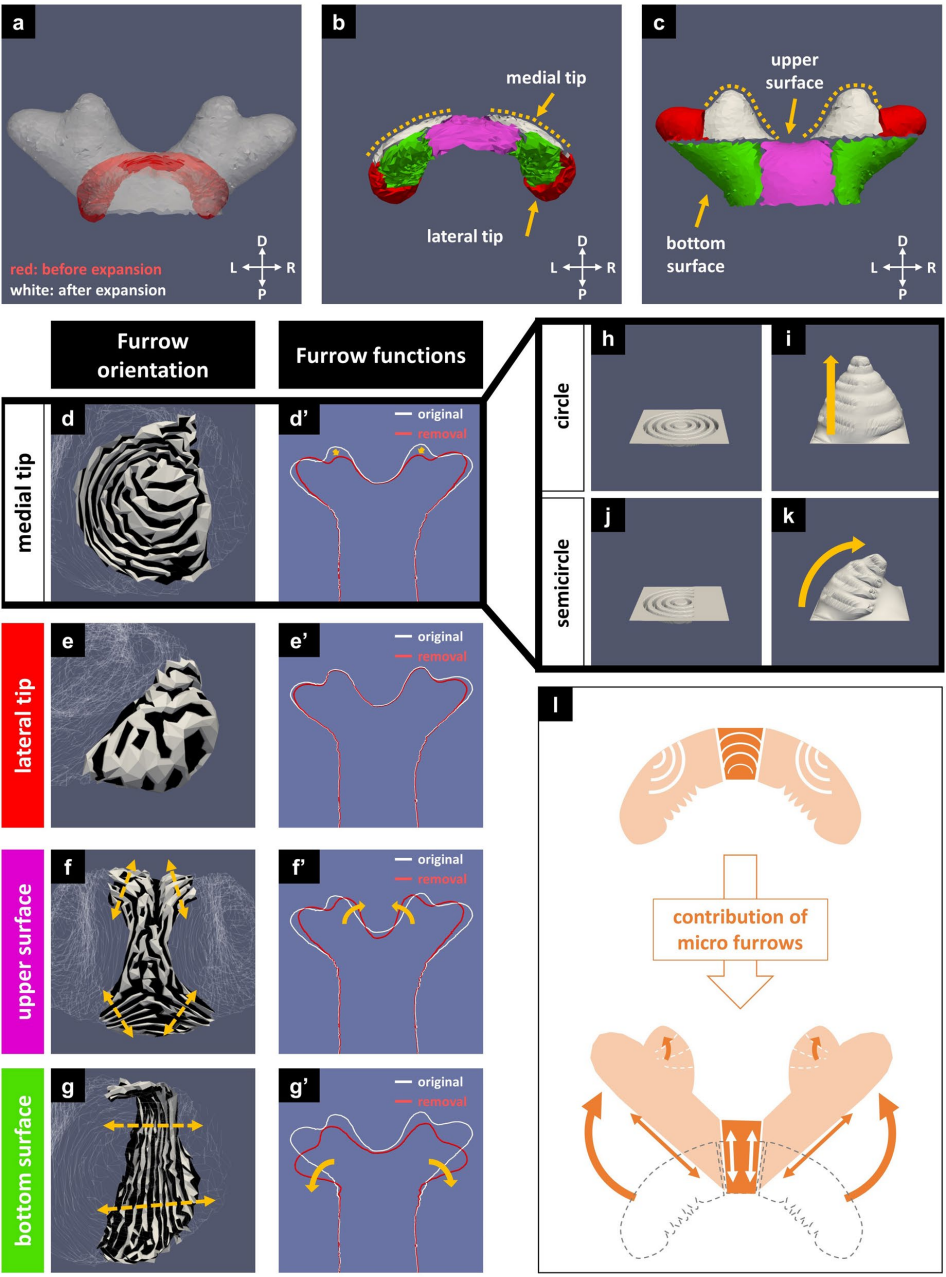
The furrow in the cap actively shapes the four-branched structure. (**a**) Superimposed drawing of the cap before and after expansion with the angle adjusted (viewed from the ventral side). The unfolded cap had a four-branched shape (white mesh), while the folded cap had a dome-like shape (red mesh). (**b**,**c**) Based on the unfolded shape, the cap was subdivided into four subregions: medial tips, lateral tips, upper surface, and bottom surface (colored in white, red, pink, and green, respectively). The area in the folded primordia corresponding to each subregion was identified in the correspondence analysis. (**d**–**g**) Furrow visualization of the cap. The direction of the yellow double-headed arrows is the estimated extending direction (perpendicular to the furrows). (**d**) In medial tips, the furrow pattern was a concentric semicircular pattern. (**e**) In lateral tips, the furrows had no distinct directionality. (**f**) The upper surface had bow-shaped furrows facing each other, like hyperbolas. (**g**) On the bottom surface, many parallel linear furrows run in the dorsoventral direction. (**d′**–**g′**) Furrow removal analysis of the cap. The difference between the cross-sections of the unfolded original mesh (white lines) and the unfolded smoothed mesh (red lines) showed the function of the furrows. (**d′**,**e′**) When the furrow pattern was removed, the medial tips became much smaller than the original ones (**d′**, yellow arrows), while the shape of the lateral tips changed little (**e′**). (**f′**,**g′**) Furrow removal on the upper surface made the angle of the central groove smaller (**f′**, yellow arrows), while that of the bottom surface made the angle of the central groove bigger (**g′**, yellow arrows). (**h**–**k**) Difference between perfect concentric circles and concentric semicircles. When expanded, the perfect circles formed a cone perpendicular to the plane (**i**), while the concentric semicircles had an oblique cone overhang (**k**). (**l**) Schematic diagram of the furrow patterns and shape change. The contribution of the furrows allows the primordial dome-like shape to transform into the pupal four-branched shape.

As mentioned above, the cap is a region that generates four branches in the distal region. Figure 5a shows a superimposed drawing of the cap before and after the expansion, with the angle adjusted to the same orientation (seen from the ventral side). We then subdivided the cap into four subregions according to the characteristics of the expanded structure (Fig. 5b,c). The lateral tips were colored red, and the medial tips were colored white. The subregion between the medial tips was named “upper surface” (colored pink) and the underside of the overhangs was named “bottom surface” (colored green). Next, the area in the folded primordia corresponding to each subregion was identified in the correspondence analysis and the functions of the furrow were examined in the furrow removal analysis.

In the medial tips, the furrow showed a concentric semicircular pattern (Fig. 5d). When the furrow pattern was removed, the medial tips became much smaller than the original ones (Fig. 5d′ and Supplementary Figure 3b′). To understand the function of the furrow pattern, we compared the concentric semicircle furrow pattern with a perfectly concentric circle pattern (Fig. 5h–k). When the simplified models were expanded, perfect circles formed a cone perpendicular to the plane (Fig. 5i), while concentric semicircular furrows had an oblique cone overhang (Fig. 5k). The angle of the medial tip was tilted outward because it was an outwardly vacant concentric semicircle. In contrast to the medial tip, the furrows in the lateral tips had no distinct directionality (Fig. 5e). The shape of the unfolded horn changed little even after the removal of the furrows (Fig. 5e′ and Supplementary Figure 3c′). This means that the morphology of the lateral tips is determined by the macroscopic shape of the horn primordia rather than the furrow pattern. On the other hand, the medial tips were not present in the folded primordia and were formed by the furrow pattern (Fig. 3b,c, yellow broken lines). Therefore, the mechanisms for the two types of tips were different.

The upper surface subregion had some bow-shaped furrows facing each other (Fig. 5f). When the furrows were removed, the angle of the central groove became smaller than the original one (Fig. 5f′ and Supplementary Figure 3d′). In the bottom surface subregion, there were many parallel linear furrows running in the dorsoventral direction (Fig. 5g). When the furrow pattern was removed in this area, the angle of the central groove became larger than the original one (Fig. 5g′ and Supplementary Figure 3e′). This transformation is explained as follows: when the parallel furrows on the bottom surface unfold, the lower surface of the overhang elongates. On the other hand, the upper surface does not elongate because the furrow pattern consists of concentric semicircles. The difference in elongation between the upper and lower surfaces raises the branches.

Thus, by using the 3D mesh data, we were able to connect the morphological changes to the furrow patterns in each part of the cap region (Fig. 5l).

## Discussion

**Figure 6:**
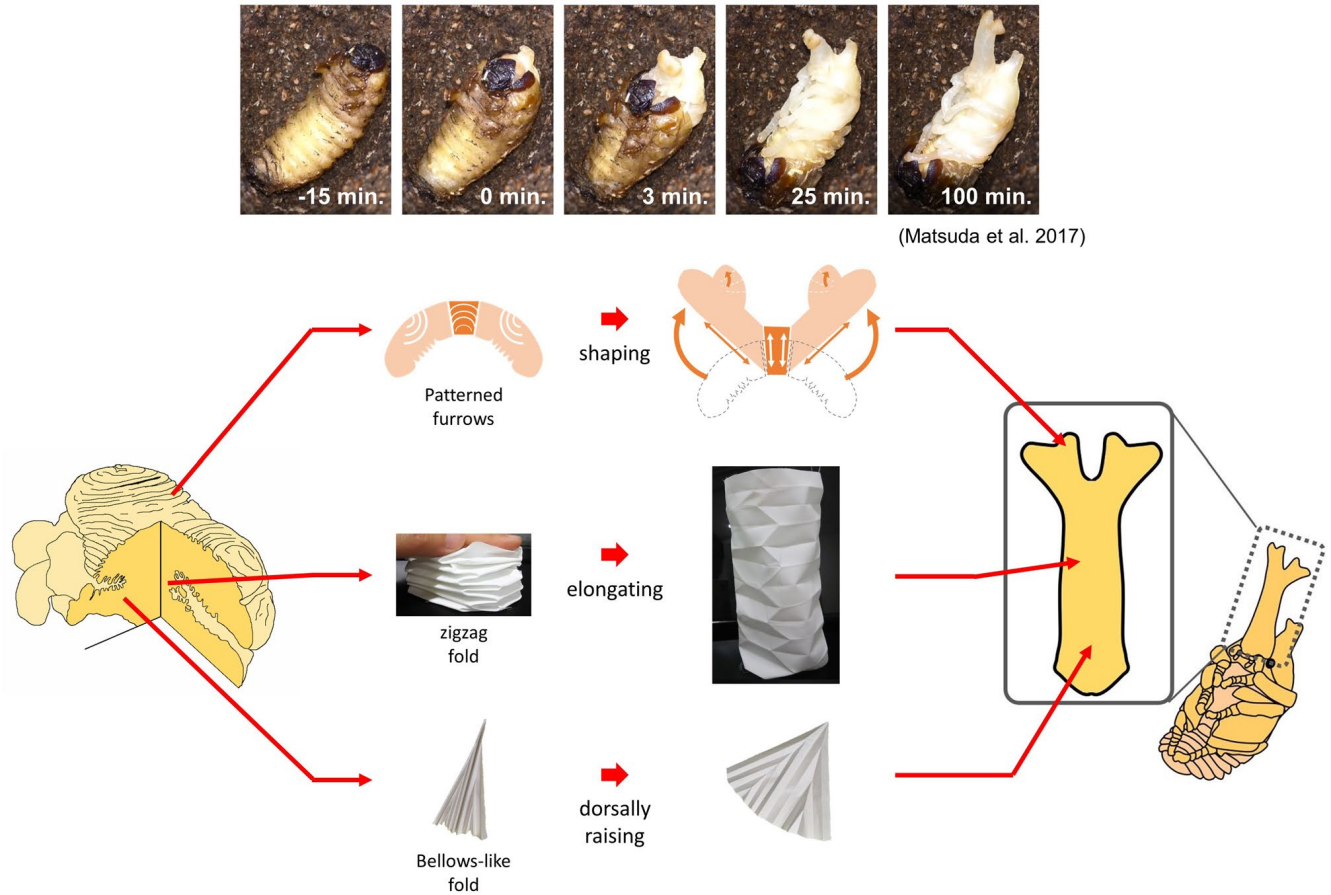
Various folding methods were employed in the biological 3D origami of the beetle horn. Transformation from horn primordium to pupal horn occurs within 2 h in vivo. During it, the folding of the horn primordium did not expand the entire primordium uniformly; different folding patterns in different positions contributed to the change of shape in different ways. The furrows in the base, whose distribution is biased to the ventral side, turn the orientation of the horn dorsally. In the cap, furrows have specific patterns, such as concentric semicircles and parallel straight patterns. These 2D patterns actively shaped the distal four-branched structure. On the other hand, the folding in the stalk showed features suggestive of passive formation. Wavy and zigzag folding may be formed by the bending force and longitudinal compression, respectively. Together, active furrows generating a specific shape and passive folding without generating any shape (essentially plane) are employed in combination to form the complex pupal horn shape.

In this study, we analyzed the 3D mesh data of beetle horn primordia with a computer simulation to decipher how the folding in horn primordia contributes to shape, size, and orientation changes during transformation. As a result, we found that the folding of horn primordia did not expand the entire primordia uniformly, but that different folding patterns in different positions contributed to shape changes in different ways (Fig. 6).

The function of furrows in the base, which connects the horn to the body, is to turn the entire horn dorsally. We found that more furrows were arranged on the ventral side than on the dorsal side, resulting in dorsal bending when unfolded. The furrow patterns in the cap were designed to create a characteristic four-branched structure at the distal tips of the Japanese rhinoceros beetle horns. The four distal tips were not created in the same way. The lateral tips were already present as the macro shape of the folded primordia. The medial tips were formed by the unfolding of concentric semicircular furrows on the upper surface of the primordia. The parallel furrows on the lower surface and the parallel bow-shaped furrows on the upper surface complementarily controlled the lifting of the lateral tips. The shape and number of horns vary between species of beetle^11,12^. Our study suggests that such variations in horn shape can be made by changing the number and pattern of furrows in each area of the cap. If the furrow pattern in the medial tips is lost, the unfolded horn changes into a bifurcated shape, which is similar to the horn of *Xylotrupes gideon.* If the furrow pattern in the medial tips is substituted for a parallel furrow pattern like on the bottom surface, the unfolded horn adopts a long-bifurcated structure similar to that of *Onthophagus mouhoti*. Most of the folding in the stalk was parallel to the tangential direction of the stalk and contributed to the elongation of the cylindrical structure. Some folding patterns seen in the stalk had features suggestive of passive formation. Because the cylindrical structure is essentially a plane, the cells composing the primordia do not need to form furrows in order to make a specific shape, but only need to maintain a zero Gaussian curvature. Therefore, the folding in the stalk might be passively formed by the growth of the cell sheet while being physically constrained.

The coexistence of “active” furrows that actively generate a specific shape and “passive” folding that does not generate any shape is one of the key findings of this analysis.

The analysis of the relationship between furrow patterns and 3D morphology can be done to some extent by experimental methods. For example, the furrow pattern can be changed by RNA interference (RNAi). However, such a method cannot precisely remove furrows in the region of interest, and cannot comprehensively trace each position during transformation. On the other hand, handling the digitized information with a computer allows us to accurately assess the impact of a furrow in any given position on the 3D morphology. The bodies of insects present various shaped projections, but most of them may be made up of a combination of folding in the stalk and furrows in the cap, as in the beetle. Therefore, we believe that the three methods used in this paper, correspondence analysis, furrow removal analysis, and furrow visualization, are useful for the study of morphogenesis by folding in other animals (see Supplementary Information 1 and 2 for details of the algorithms).

Once the relationship between furrow patterns and 3D morphology is clarified, the next challenge is to determine how to make the furrow pattern itself. Our previous paper^13^ reported that actin accumulation appears as a pre-pattern before folding patterns are formed. In addition, our recently published paper^14^ showed that the pattern and depth of horn primordial furrows are regulated by different genes. Moreover, Obst-E, a protein expressed in the cuticle of *Drosophila*, controls the formation of a stripe folding pattern, which determines the 3D shape of larvae^1^. These studies suggest that certain mechanisms actively form the folding pattern. Although the mechanisms underlying active formation remain largely unknown, some studies have reported that the patterned growth of a cell sheet in a restricted space causes various folding patterns^15,16^. A paper we recently published also showed that the genes regulating primordial furrows affect the frequency and orientation of cell divisions. Thus, the patterned growth of a cell sheet in a restricted space is the leading hypothesis explaining active furrow formation. In fact, the hypothesis may also explain passive folding formation because the passive folding of the stalk may occur as a result of the cell sheet growth in a restricted space (and the shrinkage of the restricted space). To understand the 3D morphogenesis of exoskeletal animals, it is essential to verify this hypothesis.

The purpose of this study was to elucidate the mechanisms involved in the morphogenesis of beetle horns. However, if we generalize it to the dilemma of how to confine a large 3D structure to a small space, some interesting problems emerge. The first is to study the three-dimensional shape of horn primordia mathematically as a type of packing problem. Is it possible to theoretically predict the folding method employed by the beetle (e.g., by considering the physical properties of the cell sheet and/or the contents inside the primordia)? Concerning the beetle horns, the knockdown of many genes has been reported to lead to changes in shape^8,17–24^. If the shape changes, the best folding method will also change. Whether mathematics can theoretically predict these changes is also an interesting question. The second is the industrial application of the discovered folding method. Good stuffing methods are in demand in industry, as the folding of insect wings^25–27^ has already been applied to the design of folding drones^28^. The Miura-fold, developed from the Yoshimura pattern, has also been seen in biological materials and applied to many industrial objects^29–31^. The folding of horn primordia may offer a new design method for use in the creation of large structures in limited spaces.

## Methods

### Reconstruction of virtual horn primordia from serial images

We reconstructed the virtual horn primordia using the CoMBI method^32^. The whole shape of the horn primordia is represented by a surface mesh composed of facets, edges, and vertices. After reconstruction, the mesh was processed to reduce the number of vertices and reduce noise from the mesh using Meshlab^33^. The meshes were visualized with ParaView^34^. See Matsuda et al. for details^7^.

### Unfolding of the horn primordia and correspondence analysis

To simulate the unfolding of the horn primordia, energy minimization was performed with respect to the vertex position using the steepest descent method, as described in our previous paper^7^. The numerical calculation was performed with sundials^35^. To study the correspondence, we traced each vertex position during the transformation.

Code for this software is accessible here: https://bitbucket.org/kMatsuda_klab/unfolding_mesh/src/master/.

### Removal of furrows from the mesh by smoothing algorithms

To remove furrows from the mesh, the HC-modified Laplacian smoothing algorithm was applied^36^. Applying the smoothing algorithm to a specific area enabled us to create a region-specific furrow-removed mesh. See the supplementary material for details of the algorithms and parameters.

Code for this software is accessible here: https://bitbucket.org/kMatsuda_klab/hc_laplacian/src/master/.

### Furrow visualization

To visualize the furrows, we first removed furrows from the mesh using the HC-modified Laplacian smoothing algorithm^36^. By comparing the smoothed mesh with the original mesh, the furrow patterns were colorized. See the supplementary material for details of the algorithm.

Code for this software is accessible here: https://bitbucket.org/kMatsuda_klab/furrow_visualization/src/master/.

### Micro-CT scan of horn primordia

To observe the cuticle structure clearly, we degraded the inner tissue by incubation overnight with 10% KOH solution at 60 °C, followed by washing with distilled water and dehydration with ethanol. The dehydrated sample was soaked in t-butanol and freeze-dried. Then, dried samples were scanned using a micro-CT scanner (Skyscan1172, Bruker, USA) following the manufacturer’s instructions. The X-ray source was 32 kV, and the datasets were acquired at a resolution of 9 μm/pixel. The stacks of transverse sections were reconstructed from primary shadow images using SkyScan software NRecon. From these image stacks, 3D volume-rendered images were constructed using 3D slicer software^37^.

### Analog simulations of the folding in the stalk

To simulate wavy folding, we first folded a cloth to make a single ridge. Then, this cloth was placed on a rubber block and the block was bent. The zigzag folding was simulated by pulling up the hem of the author’s pants.

## Supplementary Information

Supplementary Information 1.

Supplementary Video S1.

Supplementary Video S2.

Supplementary Video S3.

Supplementary Video S4.
