# Supplementary material for: Computational analyses decipher the primordial folding coding the 3D structure of the beetle horn
Source: Sci Rep. 2021 Jan 13;11:1017. doi: 10.1038/s41598-020-79757-2 (PMC7806817; doi:10.1038/s41598-020-79757-2)
Supplement: Supplementary file 1 — Supplementary Information 1. [file 41598_2020_79757_MOESM1_ESM.docx]

Computational analyses decipher the primordial folding coding the 3D structure of the beetle horn

Keisuke Matsuda^1,2^, Hiroki Gotoh^3^, Haruhiko Adachi^1^, Yasuhiro Inoue^4^, Shigeru Kondo^1^*

1 Graduate School of Frontier Biosciences, Osaka University, Suita, Osaka, 565-0871, Japan

2 Osaka University Hospital, Osaka University, Suita, Osaka, 565-0871, Japan

3 Ecological Genetics Laboratory, Department of Genomics and Evolutionary Biology, National Institute of Genetics, Mishima, Shizuoka, 411-8540, Japan

4 Department of Micro Engineering, Kyoto University, Kyoto, 615-8540, Japan

*Corresponding author and address. Pattern Formation Laboratory, Graduate School of Frontier Biosciences, Osaka University, Suita, Osaka, 565-0871, Japan; E-mail: skondo@fbs.osaka-u.ac.jp

## Supplementary materials

Supplementary Figure 1: Simulation of primordia unfolding.

Supplementary Figure 2: Orientation of horn primordia.

Supplementary Figure 3: Furrow removal analysis.

Supplementary Figure 4: Accordion-like folds (jabara).

Supplementary Figure 5: Origami model of the stalk.

Supplementary Information 1: Smoothing algorithm

Supplementary Information 2: Furrow visualization

Supplementary Video 1: Whole primordia

Supplementary Video 2: Cap

Supplementary Video 3: Stalk

Supplementary Video 4: Base


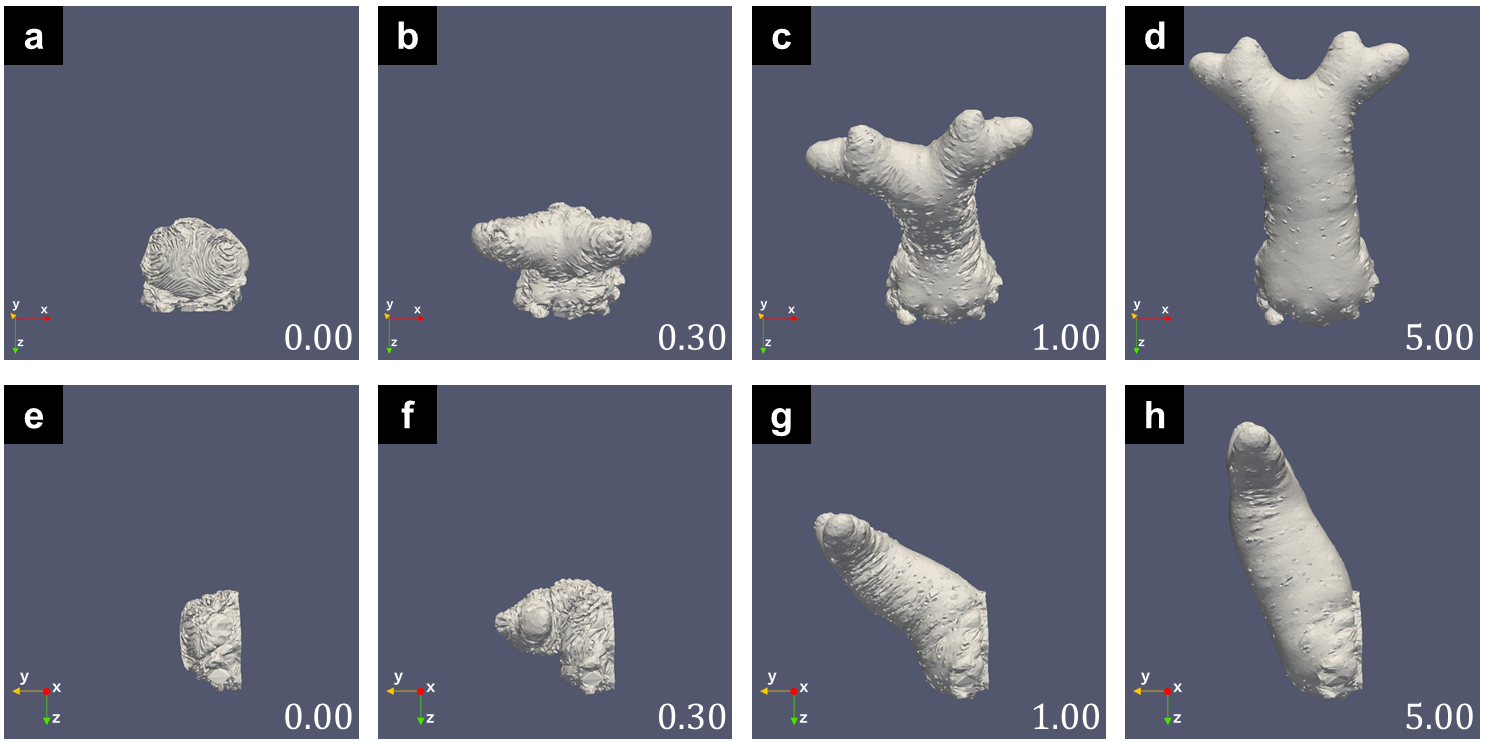


### **Supplementary Figure 1: Simulation of primordia unfolding.**

(a-h) 3D mesh data acquired with the CoMBI method was unfolded with our simulation. (a-d) Viewed from the top side of the primordia. (e-h) Viewed from the left side of the primordia.


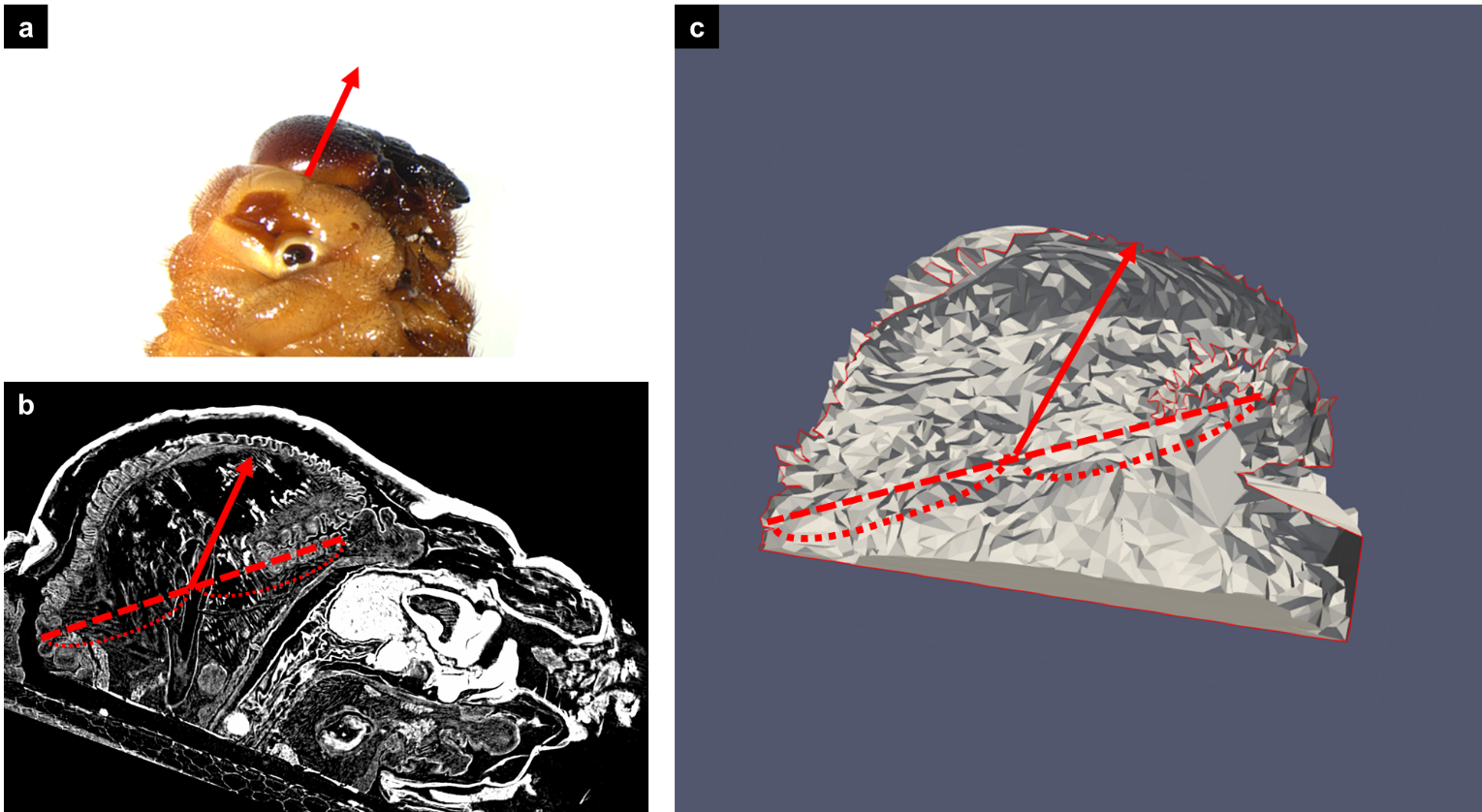


### **Supplementary Figure 2: Orientation of horn primordia.**

(a) The estimated orientation of horn primordia. The orientation of horn primordia was measured using the sagittal plane of the micro-CT data (b) and the mesh data (c). First, the middle point of the proximal end of the horn primordia was found (b, c). Next, the branching point was estimated using the mesh data (c). Then, the orientation was defined as the direction from the middle point to the branching point.


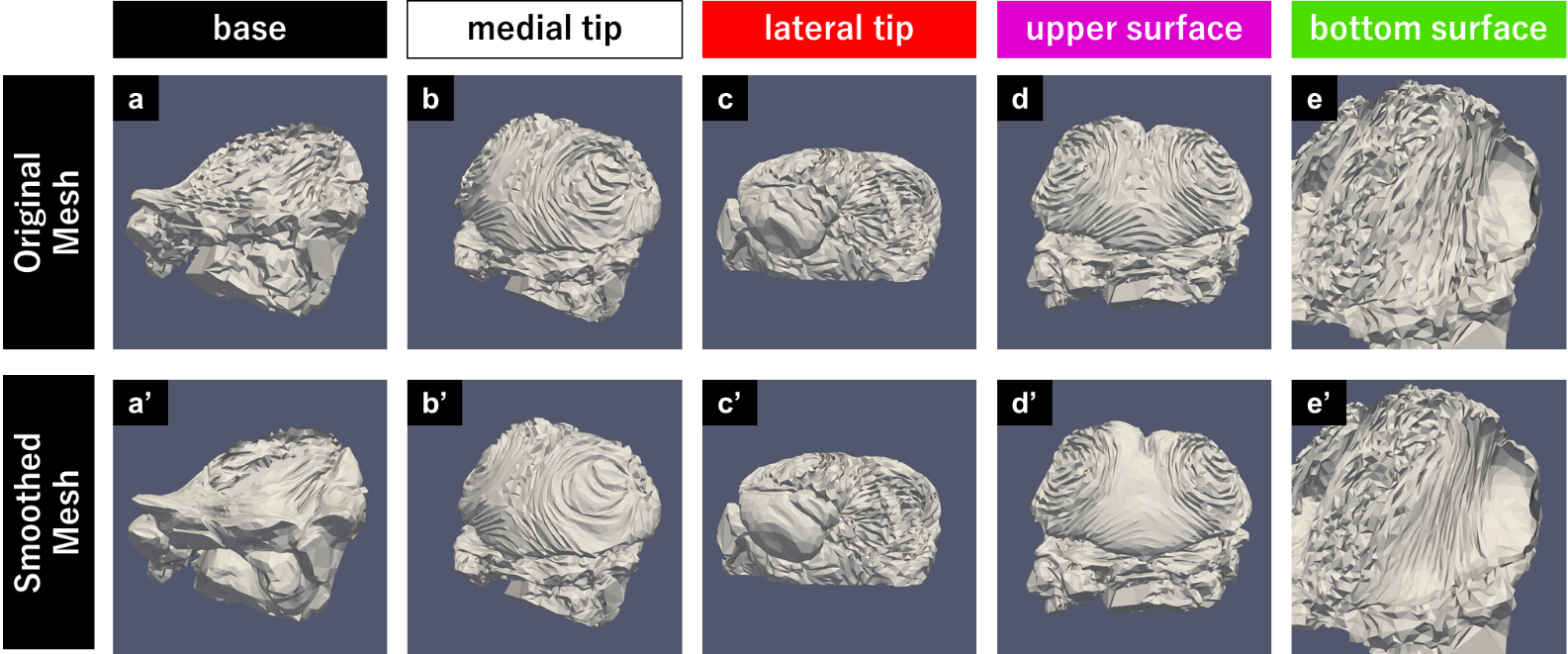


### **Supplementary Figure 3: Furrow removal analysis.**

Furrow removal analysis was performed to study the function of furrows. In the analysis, the furrows in a specific region (subregion) are shallowed by a smoothing algorithm (HC-modified Laplacian smoothing). The original meshes are shown in the upper panels (a-e) and the smoothed mesh (after shallowing furrows) are shown in the lower panels (a’-e’).


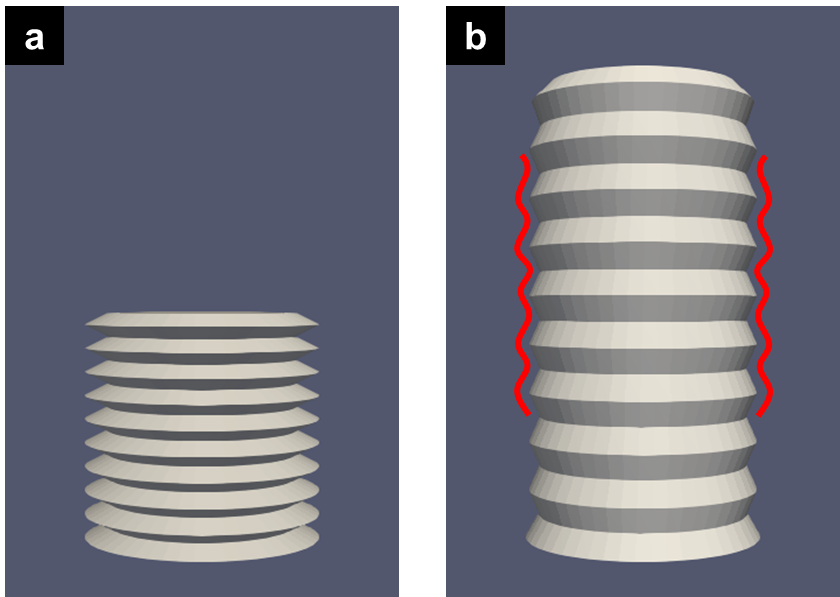


### **Supplementary Figure 4: Accordion-like folds (jabara).**

(a) Accordion-like folds are one of the simplest ways to compress a cylinder short. (b) When unfolded with the computer simulation, the mesh with accordion-like folds was still wavy.

**Supplementary Information 1: Smoothing algorithm**

To make a smoothed mesh, we applied HC-modified Laplacian smoothing^2^.

In HC-modified Laplacian smoothing, the modified point $\boldsymbol{p}_{i}$, is pushed back to the previous point $\boldsymbol{q}_{i}$ and the original point $\boldsymbol{o}_{i}$.

First, the modified point $\boldsymbol{p}_{i}$ is produced by Laplacian smoothing.

$$\boldsymbol{p}_{i}≝\frac{1}{\left| adj\left( i \right) \right|}\sum_{j\in adj\left( i \right)} \boldsymbol{q}_{i,j}$$

In the equation, $adj\left( i \right)$ denotes the set of adjacent vertices of vertex i and $\boldsymbol{x}_{i,j}$ is the position of the j-th connecting vertex.

Then, $\boldsymbol{b}_{i}$ is defined as the difference between the modified point and the previous/original point.

$$\boldsymbol{b}_{i}≝\boldsymbol{p}_{i}-\left( \alpha\boldsymbol{o}_{i}+\left( 1-\alpha\right)\boldsymbol{q}_{i} \right)$$

Finally, $\boldsymbol{d}_{i}$ is defined as the weighed average of the difference and is used for correction.

$$\boldsymbol{d}_{i}≝{\beta\boldsymbol{b}}_{i}+\frac{1-\beta}{\left| adj\left( i \right) \right|}\sum_{j\in adj\left( i \right)} \boldsymbol{b}_{j}$$

See Vollmer 1999 for more information about the algorithm.

The HC-modified Laplacian smoothing was developed to prevent shrinkage of the mesh by smoothing algorithms. To study the function of furrows, it is important not to change the macro shape of horn primordia. The parameters $\alpha$ and $\beta$, and the number of smoothing times were tuned depending on the mesh (because the fine mesh is resistant to smoothing). The parameters we used to smooth each mesh are shown in the table.

**Parameters for smoothing**

| Original mesh | Number of vertices | $\alpha$ | $\beta$ | Times of smoothing |
| --- | --- | --- | --- | --- |
| example (used in Fig.1) | 3976 | 0.0 | 0.5 | 21 |
| horn_primordia_1 | 9776 | 0.2 | 0.6 | 11 |
| horn_primordia_2 | 10474 | 0.0 | 0.5 | 21 |

**Supplementary Information 2: Furrow visualization**

To determine whether a vertex is located on a ridge or a valley, we first calculated the vertex normal for each vertex. The vertex normal of vertex i, $\boldsymbol{n}_{i}$ was calculated the following way:

1. The facet area $S_{i,j}$ and the normal vector $\boldsymbol{n}_{i,j}$ for each facet around vertex i were calculated using the smoothed mesh.

2. $\boldsymbol{sum}_{i}$ was calculated by the weighted summation of all facet normal, and $n_{i}$ was determined by normalization of $\boldsymbol{sum}_{i}$.

$$\boldsymbol{sum}_{i}=\sum_{j\in adj\left( i \right)} S_{i,j}\boldsymbol{n}_{i,j}$$

$$\boldsymbol{n}_{i}≝ \frac{1}{\left| \boldsymbol{sum}_{i} \right|}\boldsymbol{sum}_{i}$$

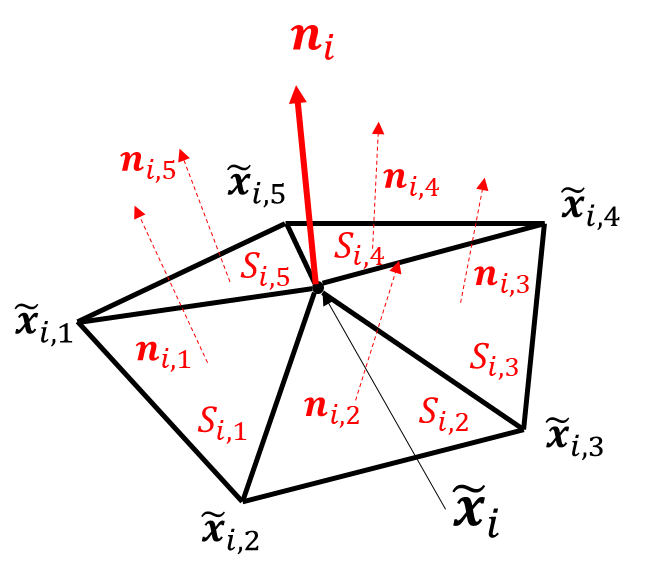


**Calculation of the vertex normal vector**

Then, the ${discriminant}_{i}$ was calculated with the following equation:

$${discriminant}_{i}≝\left( \boldsymbol{x}_{i}-{\tilde{\boldsymbol{x}}}_{i} \right)・\boldsymbol{n}_{i}$$

In the equation, $\boldsymbol{x}_{i}$ is the position of vertex i of original mesh and ${\tilde{\boldsymbol{x}}}_{i}$ is the position of vertex i of smoothed mesh.


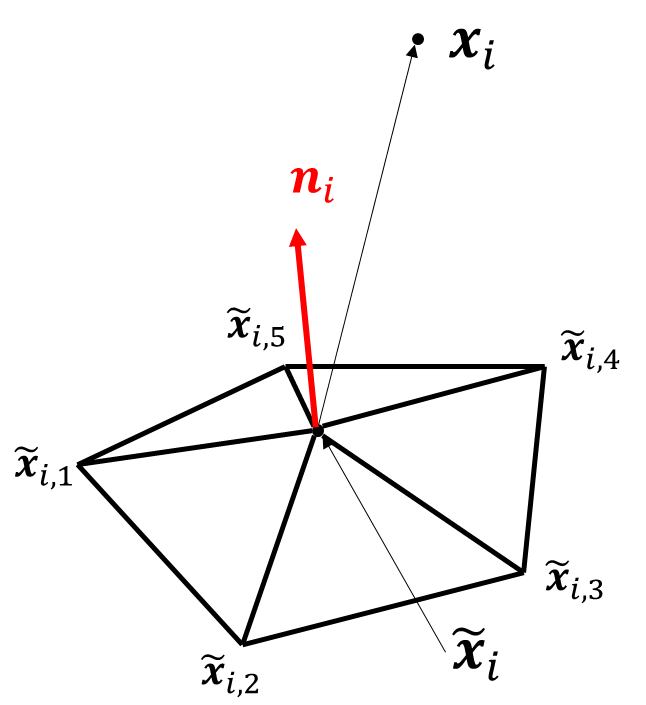


**Calculation of the discriminant**

When the ${discriminant}_{i}$ was more than 0.05, the vertex i was colorized in white (judged as “on a ridge”). When the ${discriminant}_{i}$ was less than $-$0.05, the vertex i was colorized in black (judged as “on a valley”).

**Supplementary references**

1. Ahrens, James, Geveci, Berk, Law, Charles, ParaView: An End-User Tool for Large Data Visualization, Visualization Handbook. (Elsevier, 2005) ISBN-13: 978-0123875822

2. Vollmer, J., Mencl, R. & Muller, H. Improved Laplacian Smoothing of Noisy Surface Meshes. *Computer Graphics Forum* (1999) doi:10.1111/1467-8659.00334.
